# Healthcare workers' autonomy: testing the reciprocal relationship between job autonomy and self-leadership and moderating role of need for job autonomy

**DOI:** 10.1108/JHOM-04-2022-0106

**Published:** 2022-09-07

**Authors:** Pauline van Dorssen-Boog, Tinka van Vuuren, Jeroen de Jong, Monique Veld

**Affiliations:** Open Universiteit , Heerlen, The Netherlands; Nijmegen School of Management , Institute for Management Research , Radboud University , Nijmegen, The Netherlands; Transvorm , Tilburg, The Netherlands; Loyalis , Heerlen, The Netherlands

**Keywords:** Job autonomy, Self-leadership, Healthcare workers, Cross-lagged study, Need for job autonomy

## Abstract

**Purpose:**

While both perceived job autonomy and self-leadership are assumed to be important for optimal functioning of healthcare workers, their mutual relationship remains unclear. This cross-lagged study aims to theorize and test that perceived job autonomy and self-leadership have a reciprocal relationship, which is moderated by need for job autonomy.

**Design/methodology/approach:**

Two-wave panel data were used to measure cross-lagged relationships over a time period of three months. Self-leadership is indicated by both self-leadership strategies and self-leadership behavior. The data were analyzed using hierarchical multiple regression (HMR).

**Findings:**

Job autonomy was not causally nor reverse related to self-leadership strategies, but did relate to self-leadership behavior in both directions. Need for job autonomy did not influence the causal and reverse relationships between job autonomy and self-leadership (strategies and behavior). Instead, need for job autonomy discarded the influence of job autonomy on self-leadership behavior, and predicted self-leadership behavior over time.

**Practical implications:**

For optimizing healthcare jobs, human resource management (HRM) policy makers need to consider other interventions such as training self-leadership, or developing an autonomy supportive work environment, since job autonomy does not lead to more use of self-leadership strategies.

**Originality/value:**

This study used a cross-lagged study design which gives the opportunity to investigate causal relationships between job autonomy and self-leadership. Both self-leadership strategies and self-leadership behavior are included.

## Introduction

For years, healthcare organizations and policy makers are challenged to find ways to improve the job satisfaction and health of healthcare workers ( De Lange *et al.* , 2020 ). It is broadly acknowledged that one of the important job design measures would be increasing job autonomy ( Cicolini *et al.* , 2014 ; Laschinger *et al.* , 2004 ; Broetje *et al.,* 2020 ). Job autonomy generally refers to the degree to which the job provides substantial freedom, independence and discretion to the individual in how to carry out one's job ( Hackman and Oldham, 1976 ). Research supports the idea that the perception of job autonomy contributes to the well-being of healthcare workers ( Cicolini *et al.* , 2014 ; Widerszal-Bazyl *et al.* , 2003 ; Toode *et al.* , 2011 ). Therefore, contemporary healthcare organizations increasingly seek to improve the level of job autonomy for their healthcare workers (e.g. Larsen *et al.* , 2018 ). However, scholars also suggest that not just the perception of job autonomy as characteristic of the job design, but also individual characteristics related to self-determination and autonomous functioning need to be taken into account ( Laschinger *et al.* , 2004 ; Wagner *et al.* , 2010 ). According to self-leadership theory employees can proactively take responsibility for their own motivation and performance ( Manz, 1986 ). They can take initiatives in order to organize their work load ( Lovelace *et al.* , 2007 ) and motivate and direct themselves in order to optimize their work engagement and performance ( Knotts *et al.* , 2021 ; Neck and Houghton, 2006 ; Manz, 1986 , 2015 ).

Although originally self-leadership is introduced as a substitute for external leadership ( Manz and Sims, 1980 ; Manz, 1986 ), self-leadership scholars assume that higher levels of job autonomy will provoke self-leadership ( Alves *et al.* , 2006 ; Stewart *et al.* , 2011 ). The perception of job autonomy will stimulate employees to motivate and direct themselves in order to achieve their working goals ( Müller and Niessen, 2019 ; Alves *et al.* , 2006 ). Indeed, there is some evidence that the effects of perceived job autonomy on the work engagement and health of healthcare workers can be explained by their self-leadership ( Van Dorssen-Boog *et al.* , 2020 ).

However, the Person Environment fit theory (PE fit theory) ( Kristof-Brown *et al.* , 2005 ; Caplan, 1987 ) points out that presumed relationships between job design, attitudes and behavior are mostly more complex. Employees are not just reactively responding to their work context, as they can be active designers of their job ( Frese and Fay, 2001 ; Wrzesniewski and Dutton, 2001 ). Therefore, self-leadership may help employees to make their jobs more resourceful and natural rewarding ( Stewart *et al.* , 2011 ; Neck and Houghton, 2006 ). They may attempt to organize more autonomy within their job, as job autonomy is theorized to be an important job resource for people in order to reduce work related stress and increase work engagement ( Bakker and Demerouti, 2007 ). Moreover, practicing self-leadership may give healthcare workers the experience that they actually have more autonomy in their job than priorly thought ( Lovelace *et al.* , 2007 ; Alves *et al.* , 2006 ; Bandura, 1997 ). In conclusion, we argue that the relationship between perceived job autonomy and self-leadership is bi-directional: perceived job autonomy will impact self-leadership and vice-versa. Therefore, the first aim of this study is to test this bi-directional relationship.

Additionally, self-determination theory (SDT) ( Deci and Ryan, 2000 ) argues that although people have a need for autonomy, it is not the amount of autonomy, but the satisfaction of one's need for autonomy that leads to intrinsic motivation and health ( Van den Broeck *et al.* , 2008 ). Due to multiple reasons such as formal professional responsibility ( Iliopoulou and While, 2010 ), learning experiences concerning autonomous functioning in childhood ( Schüler *et al.* , 2016 ), or individuals' need for structure ( Roberts and Foti, 1998 ), people can differ in their need for job autonomy. Hence, we propose that the effects of perceived job autonomy on one's self-leadership may be moderated by the degree to which one actually has a need for job autonomy. And vice versa, individuals' need for job autonomy will also influence the effects of self-leadership on one's perceived job autonomy, since people with higher needs for job autonomy will make an attempt to proactively improve the level of autonomy in their job. Accordingly, the second aim of this article is to investigate the moderating role of need for job autonomy on the causal and reverse relationship between perceived job autonomy and self-leadership.

The contributions of this article to the existing self-leadership and healthcare literature are threefold. First, in contrast to prior studies which only assume a causal relationship between perceived job autonomy and self-leadership (e.g. Van Dorssen-Boog *et al.* , 2020 ; Ho and Nesbit, 2014 ; Müller and Niessen, 2019 ), we hypothesize and test this bi-directional relationship by applying a cross-lagged research design. Moreover, the study includes the moderating role of need for job autonomy, as this may influence the bi-directional relationship between perceived job autonomy and self-leadership. Second, in contrast to prior self-leadership studies (e.g. Müller and Niessen, 2019 ; Yun *et al.* , 2006 ), this study includes both the measurement of self-leadership strategies and self-leadership behavior. We claim that both dimensions of self-leadership give insight in self-leadership, though from different perspectives. Self-leadership strategies refer to normative self-influencing strategies which explain how one should ideally influence own thoughts and behavior. Self-leadership behavior represents the actual self-responsible behavior at work, including taking initiative and solving problems autonomously. If research focuses on either strategies or behavior, it may lead to misinterpretation of study results as the first is a prescriptive and the second a descriptive research approach ( Elqayam and Evans, 2011 ; Van Dorssen-Boog *et al.* , 2020 ). Hence, in the present study, both perspectives are included in order to enrich our understanding of self-leadership on basis of idealism and realism. Third, this study contributes to the debate in the healthcare literature that healthcare workers need to have more job autonomy in order to be willing and able to continue working within this industry (e.g. Cicolini *et al.* , 2014 ; Broetje *et al.* , 2020 ). Drawing on PE fit theory ( Kristof-Brown *et al.* , 2005 ; Caplan, 1987 ), we argue that not every healthcare worker will equally benefit from perceived job autonomy as they individually differ in self-leadership competences as well as in their actual need for job autonomy. Moreover, we propose that self-leading healthcare workers are able to self-influence their job autonomy, and this relationship will be strengthened for those with higher needs for job autonomy. Therefore, the results of this study will help healthcare organizations, and specifically human resource management (HRM) policy makers, to develop a resourceful work environment in which people will optimally benefit from their autonomy. This may be facilitated by improving job autonomy as job design measure and also by encouraging the development of self-leadership.

## Theoretical background

### Job autonomy

Job autonomy generally refers to a characteristic of the job design, which is based on top down decisions within an organization concerning the formal decision latitude of employees. Job autonomy refers to the opportunity to autonomously plan job tasks (work scheduling autonomy), to freely decide on how to carry out given job tasks (work method autonomy), as well as the opportunity to freely choose which goals and which tasks within a job are important to do (criteria autonomy) ( Breaugh, 1999 ). According to the job design literature, jobs need to be enriched with autonomy as the self-responsibility which comes along with the job autonomy is assumed to contribute to the motivation to perform well ( Hackman and Oldham, 1976 ). Research suggests that the perception of job autonomy stimulates personal initiative at work ( Frese *et al.* , 2007 ). The increased responsibility is assumed to be motivational for proactive work behavior in order to achieve work goals ( Bakker and Demerouti, 2007 ). Moreover, perceived job autonomy is assumed to enable employees to deal with high work load ( Karasek, 1979 ; Bakker and Demerouti, 2007 ). Through the perception of job autonomy people can organize their work in such a way that it suits to their own preferences, and therefore buffers the potential negative impact of high work load on health ( Petrou *et al.* , 2012 ; Van Yperen *et al.* , 2016 ). Furthermore, perceived job autonomy is assumed to be one of the basic nutriments for intrinsic work motivation as people have a basic need for autonomy which they want to have satisfied ( Van den Broeck *et al.* , 2008 ). The perception of job autonomy will facilitate the process of self-determination and intrinsic motivation ( Van den Broeck *et al.* , 2008 ; Gagné and Deci, 2005 ). In contrast, if people perceive external control instead of autonomy in their work context, motivation will be more based on what one must do, instead of what one is willing to do, which can easily cause strain and health impairment ( Van den Broeck *et al.* , 2008 ).

## Self-leadership – strategies and actual behavior

Whereas perceived job autonomy refers to characteristics of the job design, self-leadership refers to individual's ability to autonomously motivate and direct oneself to optimal functioning ( Neck and Houghton, 2006 ; Manz, 1986 , 2015 ). Self-leadership theory proposes that employees' performances are not merely a result of the external directions and motivational pep talks by managers or colleagues, as people have the potential to motivate and direct themselves ( Manz, 1986 ; Stewart *et al.* , 2011 ). For this, self-leadership theory prescribes a broad set of cognitive and behavioral self-influencing strategies, which are based on insights from classical motivation and self-regulation theories such as SDT ( Deci and Ryan, 2000 ), self-regulation theory and self-efficacy theory ( Carver and Scheier, 1981 ; Bandura, 1977 ) and goal-setting theory ( Latham and Locke, 1991 ). *Behavioral* *focused strategies* are especially helpful, when a job task is boring, difficult or otherwise challenging, though still needs to be done. These type of strategies include self-observation, self-goal setting, self-cueing and self-reward. *Constructive thought pattern strategies* aim to manage functional patterns of habitual thinking concerning own performance. Through positive self-talk, evaluation of thoughts and assumptions and mental imagery, people can self-influence their thoughts such that these give an optimistic perspective on their ability to achieve successful performance, even in difficult, challenging situations ( Neck and Manz, 1996 ). *Natural reward strategies* aim to increase intrinsic motivation for a job ( Manz, 1986 ). One can increase natural rewards by actively creating more attractive job tasks or job conditions. Natural reward strategies also refer to changing the mental focus from unpleasant to pleasant aspects of the job. It is assumed that natural reward strategies play an important role in the process to optimal performance as they are aimed to improve intrinsic motivation for performance ( Neck and Houghton, 2006 ; Manz, 1986 ).

While the prescriptive character of self-leadership theory makes the theory highly applicable for practice, the normative strategies may also lead to a false interpretation of how self-leadership actually functions in practice ( Van Dorssen-Boog *et al.* , 2020 ). Normative theories are focused on idealized behavior, thus how one “should” behave, whereas descriptive research is aimed to observe the actual behavior ( Elqayam and Evans, 2011 ). With descriptive science the researcher “simply defines, delineates and documents its findings, leaving them free of value judgement” ( Linley *et al.* , 2006 , p. 13). We propose that self-leadership may also be reflected by the actual autonomous behavior in one's job, such as taking initiative to solve daily work related problems in an autonomous and responsible way. For this, people may not use the full range of self-leadership strategies. We explain this on basis of SDT ( Deci and Ryan, 2000 ) and social learning theory ( Bandura, 1997 ).

According to SDT, people are inherently intrinsic motivated and proactive, if their basic psychological needs for autonomy, relatedness and competence are satisfied ( Deci and Ryan, 2000 ). If employees feel satisfied with the amount of autonomy they perceive, feel competent for their job tasks and experience meaningful social relationships, this will feed their intrinsic motivation and subsequently lead to proactive work behavior ( Deci *et al.* , 2017 ). Since these people feel satisfied in their psychological needs and therefore feel intrinsic motivated for their job task, they may not be inclined to use self-leadership strategies. Furthermore, social learning theory ( Bandura, 1997 ) proposes that self-regulation and self-control processes are developed through social learning processes (e.g. observing and modeling others, getting feedback from others on own behavior). This development through social learning processes is often an unconscious process. This can be exemplified by how children develop their behavior through social learning processes ( Bandura, 1986 ). As a result of the social learning people grow their self-efficacy which also leads to actual autonomous behavior. Therefore, on basis of SDT and social learning theory, we assume that for getting insight in self-leadership, research needs to include both self-leadership strategies and the actual self-leadership behavior. We propose that self-leadership behavior will be represented by the proactive, self-responsible and autonomous functioning at work.

## Perceived job autonomy as predictor for self-leadership in healthcare

If people experience job autonomy in their jobs, meaning that they experience freedom, independence and discretion in how to carry out one's job ( Hackman and Oldham, 1976 ), they are assumed to show more self-leadership ( Stewart *et al.* , 2011 ; Alves *et al.* , 2006 ; Müller and Niessen, 2019 ). Due to the increased perceived job autonomy, employees are inclined to make more use of self-motivation and self-direction strategies in order to actually achieve their working goals ( Alves *et al.* , 2006 ; Stewart *et al.* , 2011 ). It will encourage employees to be highly reflective concerning their own functioning and to take responsibility for the quality of their work ( Stewart *et al.* , 2011 ). Besides, perceived job autonomy will give employees the opportunity to make their job more natural rewarding, by organizing their job tasks in such a way that these better suit to personal preferences ( Stewart *et al.* , 2011 ; Neck and Houghton, 2006 ).

Indeed, in a study among healthcare workers, Van Dorssen-Boog *et al.* (2020) found that perceived job autonomy is associated with the broad range of self-leadership strategies as well as self-leadership behavior. And Hornung and Rousseau (2007) investigated the effects of increased perceived job autonomy on personal initiative within a private hospital. This hospital had changed the management structure from a centralized to decentralized organization, where shared leadership was the new way of working. They surveyed 18 months and 36 months after this organization change and found that increased perceived job autonomy had long term effects on personal initiative of hospital workers. And in a study among teleworkers working in a diversity of industries, it was found that on days that employees worked from home, an increased use of self-leadership strategies, such as goal-setting and self-rewards, was reported, which was explained by increased perceived job autonomy ( Müller and Niessen, 2019 ). Based on these arguments we hypothesize the following:

H1a.
Perceived job autonomy (time 1) has a positive effect on self-leadership strategies (time 2)

H1b.
Perceived job autonomy (time 1) has a positive effect on self-leadership behavior (time 2)

## Perceived job autonomy as an outcome of self-leadership

Person Environment fit theory (PE fit theory) ( Kristof-Brown *et al.* , 2005 ) explains that relationships between job design and individual outcomes related to motivation and performance are mostly more complex, than just causal. Employees differ in their competences, needs and preferences, resulting in different dynamics between job design and employees' motivation and behavior ( Kristof-Brown *et al.* , 2005 ; Caplan, 1987 ).

People who are highly skilled in self-leadership may perceive more job autonomy as they experience that they are able to self-influence the organization of their job tasks. The competences for self-leadership will help employees to take charge of the work load and to achieve their working goals, which will give feelings of being in control in one's job ( Alves *et al.* , 2006 ; Lovelace *et al.* , 2007 ; Bakker and Demerouti, 2007 ; Bandura, 1997 ). Besides, people have a basic psychological need for autonomy which they want to have satisfied ( Van den Broeck *et al.* , 2008 ). They want to self-organize their behavior and experiences ( Deci and Ryan, 2000 ). Therefore, we assume that people will be motivated to improve their job autonomy, and self-leadership will help them to self-influence their perceived job autonomy. Therefore, we hypothesize that self-leading healthcare workers will be able to increase their perceived job autonomy:

H2a.
Self-leadership strategies (time 1) have a positive effect on perceived job autonomy (time 2).

H2b.
Self-leadership behavior (time 1) has a positive effect on perceived job autonomy (time 2).

## The moderating role of need for job autonomy

According to SDT ( Deci and Ryan, 2000 ; Gagné and Deci, 2005 ) people have a basic psychological need for autonomy, which is defined as “the need to self-organize experience and behavior, and to have activity be concordant with one's integrated sense of self” ( Deci and Ryan, 2000 , p. 231). However, it is the not the strength of the perceived autonomy, but the satisfaction of one's need for autonomy which has an effect on intrinsic motivation ( Van den Broeck *et al.* , 2008 ). SDT explains that people can differ in the amount of autonomy they prefer, and the outcomes on motivation are consequence of a fit between perceptions of autonomy and need for autonomy ( Van den Broeck *et al.* , 2008 ). According to SDT, the satisfaction of the need for autonomy is represented by the combination of the experienced freedom and independence in the social (work) context, the actual autonomous self-regulation of the individual, and the match of both these variables with individual's need for autonomy ( Deci and Ryan, 2000 ). Therefore, we propose that the potential benefits of perceived job autonomy for self-leadership will be moderated by individual's need for job autonomy.

Healthcare workers may differ in their need for job autonomy due to multiple reasons. For instance, due to a lack of professional experience, people may prefer modeling behavior and external directions above job autonomy in order to build their self-efficacy ( Bandura, 1997 ). On the other hand, within healthcare literature it is theorized that professionals such as critical care nurses, have a need for more professional autonomy in order to be able and willing to do their job task ( Iliopoulou and While, 2010 ). They sometimes feel too much medical control by physicians, and experience a mismatch between their high level of professional training and the low level of responsibility which is afforded to them ( Bucknall and Thomas, 1997 ; Iliopoulou and While, 2010 ). Besides, the strength of the need for job autonomy may also be originated in a personality characteristic which is developed through learning experiences in childhood ( Schüler *et al.* , 2016 ). If people were raised in a family and/or school in which their autonomy was highly encouraged and facilitated, they may have developed higher needs for autonomy in their later jobs. So, although autonomy is assumed to be a general nutriment necessary for human thriving, people may develop specific preferences concerning this need, which reflects “wanting autonomy” as a motivational disposition ( Schüler *et al.* , 2016 ).

Indeed, Yun *et al.* (2006) found that the need for job autonomy positively moderates the relationship between empowering leadership and self-leadership behavior, while it negatively moderates the relationship between directive leadership and self-leadership behavior. Similarly, Rietzschel *et al.* (2013) demonstrated that close external monitoring of job tasks only resulted in intrinsic work motivation and job satisfaction for people high in need for structure, which was explained by the role clarity which they experienced in their job. In contrast, people with low needs for structure, had negative effects of close monitoring of their work, related to intrinsic work motivation and job satisfaction. This was explained by the reduced perceived job autonomy. Van Yperen *et al.* (2016) found that it were not the high job demands in itself, though the combination of high job demands, with a high individual need for autonomy and a lack of perceived job autonomy which predicted the intrinsic motivation. Therefore, not every employee may equally benefit from job autonomy as not every employee has the same need for autonomy in one's job ( Yun *et al.* , 2006 ; Roberts and Foti, 1998 ; Van Yperen *et al.* , 2016 ).

In line with the arguments above, we propose that healthcare workers' need for job autonomy will influence the relationship between perceived job autonomy and self-leadership.

H3a.
Need for job autonomy (time 1) moderates the effect of perceived job autonomy (time 1) on self-leadership strategies (time 2); the effect is stronger for high levels of need for job autonomy.

H3b.
Need for job autonomy (time 1) moderates the effect of perceived job autonomy (time 1) on self-leadership behavior (time 2); the effect is stronger for high levels of need for job autonomy.

Similarly, the reverse relationship between self-leadership and perceived job autonomy will be influenced by need for job autonomy. People with higher needs for job autonomy will use their self-leadership skills to actually increase their perceived job autonomy. In addition to the above mentioned arguments, related to personality characteristics, need for job autonomy may also be rooted in deficit experiences. Sheldon and Gunz (2009) argued that need for autonomy may be higher, if people feel frustrated due to a lack of control. We assume that the effects of self-leadership on perceived job autonomy will be higher for those with higher needs for job autonomy. These people will be motivated to use their self-leadership skills for improving their job autonomy. Therefore we hypothesize the following:

H4a.
Need for job autonomy (time 1) moderates the effect of self-leadership strategies (time 1) on perceived job autonomy (time 2); the effect is stronger for high levels of need for job autonomy.

H4b.
Need for job autonomy (time 1) moderates the effect of self-leadership behavior (time 1) on perceived job autonomy (time 2); the effect is stronger for high levels of need for job autonomy.

## Research method

In this study, we take a deductive approach, in which we aim to test the (reverse) causality between job autonomy and self-leadership among healthcare workers. To achieve this, we collected quantitative data using questionnaires which we distributed among healthcare workers of Dutch healthcare organizations.

## Sample and procedure

Six Dutch healthcare organizations (two nursing homes, two organizations for disabled people, one general hospital and one military hospital) participated in this study in the year 2015. Only employees working in operational healthcare jobs were invited to join, on a voluntary basis. A group of 111 respondents filled in the questionnaire at Time 1 and of this group 95 responded on the second questionnaire three months later as well, which led to a sample of *n* = 95 for the cross-lagged study [1] . The average age of this sample at time 1 was 43.2 years (SD = 10.6) and 97% was female. Furthermore, 10.5% had completed primary/secondary school, 59% completed vocational training and 30.5% completed a college degree. This is representative for Dutch healthcare organizations, except for gender, which exceeds the percentage of female employees within Dutch healthcare (78%) ( CBS StatLine, 2017 ). Around 56% worked within care for disabled people, 29% worked within a nursing home and 15% worked within a hospital. Respondents worked in operational jobs as support worker (40%), assistant support worker (5%), nurse in nursing home (10%), care assistant in nursing home (8%), care coordinator in disability care or nursing home (16%), registered hospital nurse (14%) and 7% had a different healthcare job, including job coach for clients, activity worker, speech therapist pharmacy assistant or laboratory assistant [2, 3] .

## Measurement instruments

*Perceived job autonomy.* Perceived job autonomy was measured with the nine-item scale for job autonomy as designed by Morgeson and Humphrey (2006) . Items refer to decision making autonomy (three items, e.g. “The job allows me to make a lot of decisions on my own”), work scheduling autonomy (three items, e.g. “The job allows me to decide on the order in which things are done on the job”) and work method autonomy (three items, e.g. “The job allows me to make decisions about what methods I use to complete my work”).

*Self-leadership* *behavior* For measuring self-leadership behavior, we used the six-item self-leadership scale as used by Yun *et al.* (2006) , which is based on the preliminary work by Cox (1993) . Example items of this scale are “I solve problems when they pop up, without always getting my supervisor's stamp of approval”, “I take initiatives on my own” and “I assume responsibilities on my own”.

*Self-leadership strategies* For measuring self-leadership strategies we selected seven full subscales of the Revised Self-leadership Questionnaire ( Houghton and Neck, 2002 ). For measuring behavior-focused strategies we used the subscales “Goal setting” (five items, e.g. “I establish specific goals for my own performance”), “Self-observation” (four items, e.g. “I make a point to keep track on how well I am doing at work”), “Self-rewards” (three items, e.g. “When I have successfully completed a task, I often reward myself with something I like”) and “Self-cueing” (two items, e.g. “I use written notes to remind myself of what I need to accomplish”). For measuring constructive thought pattern strategies we took the subscale for “Evaluation of thoughts and assumptions” (four items; “I try to mentally evaluate the accuracy of my own beliefs about situations I am having problems with”) and self-talk (three items; “Sometimes I talk to myself (out loud or in my head) to work through difficult situations”). For measuring natural rewards strategies we used the natural rewards strategies scale (five items, e.g. “I focus my thinking on the pleasant rather than the unpleasant aspects of my job activities”).

*Need for job autonomy.* Need for job autonomy was assessed using a five-item-measure that was developed for the current study, which was inspired by the job autonomy scale by Morgeson and Humphrey (2006) . The following five items were included: “I have the need to decide independently on the order in which things are done on the job,” “I have the need to make my own decisions about how to schedule my work,” “I have the need to be able to make decisions about my work on my own,” “I have the need to independently solve work-related problems” “I have the need to independently determine how I do my work”.

All scales were measured on a five-point Likert scale ranging from strongly disagree (1) to strongly agree (5).

For all the scales we checked the factor structure with CFAs in Mplus ( Muthén and Muthén, 2017 ). Due to the small sample size we tested the factor structures of each of the main study variables separately. For RMSEA the norm is < 0.05 for a good model fit and <0.08 for an acceptable model fit, and for CFI the norm is > 0.95 for a good model fit and values between 0.90 and 0.95 for an acceptable model fit. SRMR needs to stay below 0.10 ( Schweizer, 2010 ). In line with theory we found a good model fit for perceived job autonomy on the basis of three sub-dimensions ( *X* ^*2*^ *(df* *=* *24)* = 15.06, *p* > 0.05; RMSEA = 0.00; CFI = 1.00; SRMR = 0.03). Self-leadership behavior also had a satisfactory model fit on the basis of one factor ( *X* ^*2*^ *(df* *=* *9)* = 10.56, *p* > 0.05; RMSEA = 0.05; CFI = 0.98; SRMR = 0.05). Need for job autonomy needed a two-factor structure, leading to an acceptable model fit, except for RMSEA ( *X* ^*2*^ *(df* *=* *4)* = 3.62, *p* > 0.05; RMSEA = 0.00; CFI = 1.00; SRMR = 0.04). For self-leadership we found low estimates for the latent factor self-talk on self-leadership (second order factor), leading to an unacceptable model fit. Self-talk might have been too specific for respondents, and therefore not fitting to other self-leadership strategies. We decided to delete self-talk from the self-leadership scale, which led to an acceptable model fit with six latent variables ( *X* ^*2*^ *(df* *=* *203)* = 283.79 (203); *p* < 0.001; RMSEA = 0.07; CFI = 0.86; SRMR = 0.09).

## Analysis strategy

The primary interest in this study is testing our hypotheses at the individual level of analysis, i.e. testing the relationship between perceived job autonomy and individual skills, needs and behavior. However, since our respondents are employed within different healthcare organizations we controlled for organization as a determinant for our dependent variables. We controlled for organization by regressing five dummy variables on our outcome variables. None of the dummies were found to be significant [3] .

Data were analyzed using SPSS (version 25). We first tested normality, linearity, homoscedasticity and multicollinearity. We checked the reliability of the scales by checking Cronbach's alpha at each time point and by checking inter-correlations between the same constructs over time. We expect that our constructs will be quite stable, with alphas above 0.70 and expect stronger correlations (above 0.50) between the same constructs over time, with only small fluctuations.

Hypotheses were tested by conducting hierarchical multiple regression (HMR) analyses. We controlled in step 1 for the dependent variables at time 1 (e.g. in case that job autonomy T2 was the dependent variable we included perceived job autonomy T1 in the first step). In order to prevent multicollinearity in the regression analysis, centered scores were used in the regressions in which interaction effects with need for job autonomy were involved.

## Results

### Descriptive analysis

Descriptive statistics ( Table 1 ) showed that all variables slightly increase in their mean from T1 to T2. Additional T-tests demonstrated that this increase was significant for self-leadership strategies ( *p* < 0.01) and perceived job autonomy ( *p* = 0.012). At T1 we did not find significant cross-sectional relationships between perceived job autonomy and self-leadership (both strategies and behavior), while at time 2 the cross-sectional relationships between perceived job autonomy and self-leadership were significant for both strategies and behavior. Need for job autonomy T1 and T2 had small to medium significant cross-sectional relationships with both perceived job autonomy and self-leadership (both strategies and behavior). Cronbach's alphas were stable and above 0.72 over time.

## Testing hypothesized models

Four HMRs were used to test our hypotheses ( Tables 2 and 3 ). We controlled for age, but this did not influence our results. Considering the size of the sample we decided to report the most parsimonious model. Our first set of hypotheses was aimed to test whether perceived job autonomy has a cross-lagged effect (three months) on self-leadership strategies ( Hypothesis 1a ) and self-leadership behavior ( Hypothesis 1b ). After controlling for baseline levels of the dependent variables, we could not confirm this proposed relationship for self-leadership strategies. However, for self-leadership behavior we found a small effect ( *β* *= 0* . *17; p* *< 0* . *05* ). Hypothesis 1a is rejected and hypothesis 1b is accepted.

In our second set of hypotheses a reverse effect between self-leadership strategies and perceived job autonomy ( Hypothesis 2a ) and self-leadership behavior and perceived job autonomy ( Hypothesis 2b ) was expected. We did not find a reversed effect of self-leadership strategies on job autonomy, but we found a small effect for self-leadership behavior ( *β* *= 0* . *29; p* *< 0* . *001* ). Hence, hypothesis 2a is rejected, while hypothesis 2b is accepted.

In our third set of hypotheses we expected an interaction effect of need for job autonomy on the relationship between perceived job autonomy and self-leadership strategies ( Hypothesis 3a ) and self-leadership behavior ( Hypothesis 3b ). For both self-leadership strategies and self-leadership behavior there was no effect, resulting in a rejection of hypotheses 3a and 3b. However, it became clear that need for job autonomy (T1) directly influences the self-leadership behavior of healthcare workers at T2 ( *β* *= 0* . *21; p* *< 0* . *05* ). Moreover, in the third step of the hierarchical regression perceived job autonomy was no longer a significant predictor for self-leadership behavior, which implicates that need for job autonomy (T1) is a stronger predictor for self-leadership behavior (T2), than perceived job autonomy (T1).

Lastly, the moderating influence of need for job autonomy on the effect of respectively self-leadership strategies ( Hypothesis 4a ) and self-leadership behavior ( Hypothesis 4b ) on perceived job autonomy was tested. Again, the interaction effect was not found and therefore also hypothesis 4a and 4b were rejected.

## Discussion

The present study tested the causal and reverse relationships between perceived job autonomy and self-leadership (strategies and behavior) among healthcare workers. Moreover, it was hypothesized that individual's need for job autonomy will positively influence these relationships. Despite our expectations, perceived job autonomy did not predict self-leadership strategies, nor predicted self-leadership strategies perceived job autonomy. Yet, perceived job autonomy was associated with self-leadership behavior and vice versa self-leadership behavior predicted perceived job autonomy. The hypothesized moderating role of need for job autonomy on both the causal and reverse relationship between perceived job autonomy and self-leadership (strategies and behavior) was not confirmed as well. In fact, need for job autonomy directly predicted self-leadership behavior, and made the influence of perceived job autonomy on self-leadership behavior insignificant.

## Theoretical implications

While we theorized a causal and reverse relationship between perceived job autonomy and self-leadership, we could only partly confirm this. We found a small reciprocal relationship between perceived job autonomy and self-leadership behavior, but not between perceived job autonomy and self-leadership strategies. Apparently, within our sample of healthcare workers, perceived job autonomy does influence the autonomous behavior, but it does not encourage the use of self-influencing strategies.

We can think of several reasons for these outcomes. First, autonomous working healthcare workers may have less need for using self-leadership strategies as proposed by self-leadership scholars, as their jobs may be clearly defined and practical rather than conceptual ( Konradt *et al.* , 2009 ; Manz, 2015 ). As such job autonomy may encourage to some extend the autonomous self-leadership behavior, while people are not using strategies such as self-observation, goal-setting and evaluation of thoughts and assumptions. Ugurluoglu *et al.* (2015) adds to that as they found that more experienced and older healthcare workers have a reduced use of self-leadership strategies as compared to their younger colleagues. They may lead themselves on basis of the many years of experience, and have less need for using self-leadership strategies. According to social learning theory, vicarious experiences help people to exercise control, which subsequently leads to experience of having influence ( Bandura, 1997 ).

Second, there may be other variables which influence both perceived job autonomy and self-leadership strategies, for instance the autonomy support of managers and co-workers. Organizations which support the autonomous functioning of healthcare workers, may not only increase the job autonomy, but also implement management development programs which help managers to develop an empowering leadership style. Empowering leadership involves power sharing, motivation support and development support, and is intended to positively influence autonomous working. Indeed, Amundsen and Martinsen (2015) found that empowering leadership contributes to the self-leadership of followers. Furthermore co-workers can also support each other in their autonomous functioning. If people give autonomy support they actively provide opportunities for choice and self-initiation, and consider the perspective of the other person ( Baard *et al.* , 2004 ). Moreover, being autonomy supportive means that one acknowledges the feelings of the other, use non-controlling language and offers choices ( Jungert *et al.* , 2021 ). Recently, Fernet *et al.* (2021) have shown that both supervisors and coworkers can support the autonomy and as such influence nurses' autonomous motivation for work.

Thirdly, the relationship between perceived job autonomy and self-leadership strategies may especially be manifest in case of the experience of high work load. The healthcare literature assumes by referring to job demand control model ( Karasek, 1979 ) and job demands resources model ( Bakker and Demerouti, 2007 ) that the experience of job autonomy can buffer the effects of high work load on outcomes related to well-being ( Broetje *et al.,* 2020 ; Laschinger *et al.* , 2001 ). Especially in case of high work load, people may use self-leadership strategies in order to gain control in the job ( Lovelace *et al.,* 2007 ). In contrast, if healthcare workers feel comfortable with the work load, perceived job autonomy may lead to autonomous work behavior, while people feel no need for using the self-leadership strategies. Thus, there may be a three-way interaction effect in which high work load will interact with self-leadership strategies and job autonomy.

Fourth, active development of self-leadership through training, may interact with the relationship between perceived job autonomy and self-leadership strategies. Kubicek *et al.* (2014) found that although job autonomy contributes to the work engagement and mental well-being of healthcare workers in nursing homes, the experience of too much job autonomy can reduce the work engagement and mental well-being. They proposed that it may help to give nurses the opportunity to develop their competences for taking control in their jobs, for instance through job crafting interventions. Indeed, Van Wingerden *et al.* (2016) showed that a job crafting intervention among healthcare professionals with the aim to optimize their job demands and resources, and to increase their psychological capital (i.e. self-efficacy, hope, optimism and resilience), results in better work engagement and performance. Besides, also the training of self-leadership helps healthcare workers to improve their self-leadership competences, resulting in better wellbeing and performance ( Van Dorssen-Boog *et al.* , 2021 ; De Lange *et al.* , 2021 ). This implicates that it is worthwhile to facilitate the training of self-leadership, especially if healthcare professionals are expected to function autonomously in their jobs.

Interestingly, individual's need for job autonomy did not interact with the relationships between perceived job autonomy and self-leadership. We expected on basis of SDT that if people have higher needs for job autonomy this will influence the effects of perceived job autonomy on self-leadership, and will also stimulate self-leaders to gain more job autonomy. Also other research studies on the moderation effect of need strength on motivation and organization behavior find minor evidence for this assumption (e.g. Wörtler *et al.* , 2020 ). Perhaps, if we included work load in the research, it would have influenced our results, as Van Yperen *et al.* (2016) found that the positive effects of perceived job autonomy under condition of high work load, especially count for those with higher needs for job autonomy.

## Strengths and limitations

Our two-wave panel research is among one of the first studies examining the cross-lagged relationship between perceived job autonomy and self-leadership. Until now, this presumed relationship was mainly investigated cross-sectional, which can easily lead to misinterpretation of causality. Another strength of this study is that our sample included respondents from six different healthcare organizations, which gives us an indication for the generalizability of our results, especially within the healthcare industry.

However, the current study also has limitations. Since our sample size was small, we might have been unable to find significant effects, since the power of cross-lagged studies with a moderate size (between 75 and 300) is known to be low ( Kenny, 1975 ). However, as we used not more than five independent variables in our regression, we strictly followed guidelines for multiple regression methods and reduced risk for type 2 error ( Tabachnick and Fidell, 2014 ). Furthermore, our timeframe of three months might not have been accurate for measuring an effect of perceived job autonomy on self-leadership, as related studies found effects over time periods of at least a year (e.g. Frese *et al.* , 2007 ). Nevertheless, the current study adds to theory building around the proposed relationship between job autonomy and self-leadership, as we specifically focused on the cross-lagged relationship between job autonomy and self-leadership over a time period of three months.

Lastly, for this study self-reports were used, which might have led to socially desirable answers and to common method variance ( Podsakoff *et al.* , 2003 ). However, observation of self-leadership by other respondents, like supervisors or colleagues, is expected to be inaccurate, as the self-leadership process is mainly an internal process which takes place within persons ( Conway and Lance, 2010 ). To reduce the risk of consistency motives we separated the administration of the questionnaires by three months between T1 and T2 to reduce the ability of the respondent to recall previous answers on the questionnaire ( Spector, 2006 ). Furthermore, we adjusted a set of procedural strategies. For clarity of the constructs, we created a psychological separation between the measures in the questionnaire. To reduce socially desirable answers, respondents were assured that their responses were used for scientific purposes only, and were not shared with their employer. Moreover, respondents were encouraged to fill in their questionnaire as honestly as possible.

## Future research

Although the current study shed some light on the cross-lagged relationship between perceived job autonomy and self-leadership, Mitchell and James (2001) state that causal relationships might have more complex patterns. They recommend to not only think about the time period that is needed for A to cause B, but also about the duration of A before it causes B, as well as the sustainability of the effect. This implies that more measurements in time are needed in order to be able to include both change, effects and duration of effects. Moreover, different time periods should be included, in order to test how long it takes for A to cause B, and how long the supposed effect sustains. For instance, Frese *et al.* (2007) showed with a longitudinal study over a period of 4 years with measurements in every year, that it took four years to find effects of personal initiative on job autonomy. Moreover, they were able to test the mediating effects of another variable (control orientation), which helps to uncover the explaining factors in the relationship. Longitudinal research with multiple measurements over time would give insight in the time which is needed for respectively job autonomy and self-leadership to have an effect on each other. Moreover, the inclusion of other influencing factors such as individuals need for job autonomy, specific interventions, or perceived work load can help to better understand why and how job autonomy and self-leadership are related.

Furthermore, the fact that different results were found for self-leadership strategies and behavior underpins the relevance of including both measurement instruments in self-leadership research. In the study by Van Dorssen-Boog *et al.* (2020) it was found that self-leadership behavior, cognitive and behavioral strategies and natural rewards strategies have different relationships with work engagement and general health of healthcare workers. It is worthwhile to further investigate how self-leadership strategies and self-leadership behavior are mutually related and how they influence outcomes related to motivation and performance.

## Practical implications

For optimizing the autonomous functioning of healthcare professionals, healthcare organizations need to consider other interventions, than just increasing job autonomy. The present study showed that job autonomy does not predict the utilization of self-leadership strategies within a time frame of three months. Moreover, the influence of perceived job autonomy on self-leadership behavior disappears if individual's own need for job autonomy is included as predictor. Much more time may be needed for job autonomy to have an effect on individual's self-leadership (e.g. Frese *et al.* , 2007 ; Hornung and Rousseau, 2007 ). Besides, this study also showed that self-leadership strategies have no influence on perceived job autonomy in a three months' time frame. Only self-leadership behavior influences perceived job autonomy. However, research suggests that job autonomy is an important job resource for employees as it can satisfy their basic psychological need for autonomy ( Van den Broeck *et al.* , 2008 ), while job autonomy also appeals for self-leadership, as employees need to be able to solve their work related problems in a more self-responsible and independent way ( Kubicek *et al.* , 2014 ). Therefore, healthcare organizations need to think how they can more actively promote self-leadership development within their organization. We suggest several avenues for developing self-leadership.

First, organizations can improve self-leadership of healthcare professionals through offering intervention programs related to the development of self-leadership (e.g. Van Dorssen-Boog *et al.* , 2021 ; De Lange *et al.* , 2021 ; Van Wingerden *et al.* , 2016 ), as this will directly help healthcare professionals to autonomously take control in their job demands and influence their well-being. Second, organizations can investigate whether healthcare professionals need extra support in their professional development, in order to feel competent for doing their jobs autonomously ( Pursio *et al.* , 2021 ). Third, organizations may utilize the opportunities for social learning processes in the development of self-leadership ( Bandura, 1997 ). Supervisors can inspire healthcare professionals by modeling self-leadership by themselves, and also by practicing an empowering leadership style. Through empowering leadership supervisors can actively invite employees to participate in decision making, and to share their personal ideas about how to solve daily problems ( Amundsen and Martinsen, 2015 ). Besides, also co-workers can be trained in how they can coach each other with the reflection on personal values and goals, and encourage each other to act on basis of autonomous motivation ( Fernet *et al.* , 2021 ; Jungert *et al.* , 2021 ; Van Dorssen-Boog *et al.* , 2019 ). Moreover, healthcare teams may conjointly develop self-leadership, as this will help them to implement and secure the utilization of self-leadership strategies in their daily work ( Fernet *et al.* , 2021 ; Van Dorssen-Boog *et al.* , 2019 ). Team members can learn how self-leadership can help them to conjointly take control in reducing the work load and improving work engagement within their team ( Van Dorssen-Boog *et al.* , 2019 ). Lastly, the organization needs to pay attention for building a positive learning climate in which people feel allowed to practice self-leadership ( Van Vuuren *et al.* , 2016 ). For this, HRM also needs to extensively communicate the opportunities for personal and professional development, as this will help employees to decide whether or not to participate in specific intervention programs.

To conclude, for developing the autonomous functioning of healthcare professionals, healthcare organizations need to consider other interventions than just enhancing job autonomy, as we found that job autonomy only has a reciprocal relationship with self-leadership behavior, but not with self-leadership strategies.

## Figures and Tables

**Table 1:** Descriptives - mean, standard deviation, skewness, kurtosis, correlations, cronbachs' alpha (diagonal)

	Mean	SD	Skewness	Kurtosis	1	2	3	4	5	6	7	8	9
1. Age	43.17	10.59	−0.20	−1.07									
2. Job autonomyT1	2.86	0.61	−0.11	−0.86	0.00	*0* . *90*							
3. Job autonomyT2	3.02	0.71	−0.24	−0.25	−0.02	0.61^**^	*0* . *93*						
4. SL behavior T1	3.59	0.50	−0.26	−0.55	0.14	0.14	0.36^**^	*0* . *77*					
5. SL behavior T2	3.67	0.57	−0.56	−0.27	0.01	0.25^*^	0.59^**^	0.58^**^	*0* . *81*				
6. SL strategies T1	3.00	0.44	−0.36	−0.03	−0.23*	0.13	0.18	0.33^**^	0.37^**^	*0* . *84*			
7. SL strategies T2	3.12	0.49	−0.10	−0.12	−0.15	0.18	0.40^**^	0.29^**^	0.55^**^	0.73^**^	*0* . *72*		
8. Need for JA T1	3.36	0.59	−0.32	0.59	0.12	0.27^**^	0.25^*^	0.35^**^	0.41^**^	0.21^*^	0.26^*^	*0* . *79*	
9. Need for JA T2	3.42	0.59	0.26	0.53	0.01	0.16	0.38^**^	0.29^**^	0.54^**^	0.20	0.36^**^	0.46^**^	*0* . *79*

**Note(s):** * *p* < 0.05, ** *p* < 0.01, *** *p* < 0.001

**Table 2:** Results for hierarchical regression with self-leadership T2 (strategies and behavior) as dependent variable ( *n* = 95)

Step	Predictors	*F* value	Adj. *R* ^*2*^	Β (standardized)	*R* ^*2*^ change	*F* change
	*Self-leadership strategies T2*					
1	Model 1	105.54***	0.52		0.53	105.54***
	Self-leadership strategies T1			0.73***		
2	Model 2	53.85***	0.53		0.01	1.55
	Self-leadership strategies T1			0.72***		
	Job autonomyT1 (centr)			0.09		
3	Model 3	27.21***	0.53		0.01	0.80
	Self-leadership strategies T1			0.70***		
	Job AutonomyT1 (centr)			0.07		
	Need for job autonomy T1 (centr)			0.09		
	Job Aut *Need for job aut T1(centr)			−0.02		
	*Self-leadership behavior T2*					
1	Model 1	47.1***	0.33		0.34	47.1***
	SL behavior T1			0.58***		
2	Model 2	26.39***	0.35		0.03	4.1*
	SL behavior T1			0.56***		
	Job AutonomyT1 (centr)			0.17*		
3	Model 3	14.91***	0.37		0.03	2.5
	SL behavior T1			0.49***		
	Job AutonomyT1 (centr)			0.12		
	Need for job autonomy T1 (centr)			0.21*		
	Job Aut *Need for job aut T1 (centr)			0.02		

**Note(s):** * *p* < 0.05, ** *p* < 0.01, *** *p* < 0.001

**Table 3:** Results for hierarchical regression with job autonomy T2 as dependent variable ( *n* = 95)

Step	Predictors	*F* value	Adj. *R* ^*2*^	Β (standardized)	*R* ^*2*^ change	*F* change
	*Self-leadership strategies T1*					
1	Model 1	53.87***	0.36		0.37	53.9***
	Job autonomy T1			0.61***		
2	Model 2	27.73***	0.36		0.01	1.38
	Job autonomy T1			0.59***		
	SL strategies T1 (centr)			0.10		
3	Model 3	13.83***	0.35		0.01	0.33
	Job autonomy T1			0.58***		
	SL strategies T1 (centr)			0.09		
	Need for job autonomy T1 (centr)			0.07		
	SL strategies *Need for job aut T1 (centr)			0.01		
	*Self-leadership behavior T1*					
1	Model 1	53.87***	0.36		0.37	53.9***
	Job autonomy T1			0.61***		
2	Model 2	36.61***	0.43		0.08	12.6***
	Job autonomy T1			0.57***		
	SL behavior T1 (centr)			0.28***		
3	Model 3	18.12***	0.42		0.00	0.24
	Job autonomy T1			0.56***		
	SL behavior T1 (centr)			0.28***		
	Need for Autonomy T1 (centr)			−0.01		
	SL behavior *Need for job aut T1 (centr)			−0.06		

**Note(s):** * *p* < 0.05, ** *p* < 0.01, *** *p* < 0.001

## Notes

1. Additional analysis ( *t* -tests) showed that whether respondents dropped out was random

2. We used ANOVA's to test whether professions and organizations led to differences with respect to perceived job autonomy, need for job autonomy and self-leadership (strategies and behavior). No group differences were found.

3. The regression results are available upon request from the first author.
